# Factors influencing access to and utilisation of youth-friendly sexual and reproductive health services in sub-Saharan Africa: a systematic review

**DOI:** 10.1186/s12978-021-01183-y

**Published:** 2021-06-27

**Authors:** Lesley Rose Ninsiima, Isabel Kazanga Chiumia, Rawlance Ndejjo

**Affiliations:** 1 Department of Health Systems and Policy, College of Medicine, The University of Malawi, Blantyre, Malawi; 2 Department of Disease Control and Environmental Health, School of Public Health, College of Health Sciences, Makerere University, Kampala, Uganda; 3 Africa Center of Excellence in Public Health and Herbal Medicine, Department of Health Systems and Policy, Global Health Implementation, University of Malawi, Blantyre, Malawi

**Keywords:** Adolescents, Barriers, Facilitators, Reproductive health, Youth, Africa

## Abstract

**Background:**

Despite the global agreements on adolescents’ sexual and reproductive health and rights, access to and utilisation of these services among the youth/adolescents remain unsatisfactory in low- and middle-income countries which are a significant barrier to progress in this area. This review established factors influencing access and utilisation of youth-friendly sexual and reproductive health services (YFSRHS) among the youth in sub-Saharan Africa to inform programmatic interventions.

**Methodology:**

A systematic review of studies published between January 2009 and April 2019 using PubMed, Web of Science, EMBASE, Medline, and Cochrane Library, and Google Scholar databases was conducted. Studies were screened based on the inclusion criteria of barriers and facilitators of implementation of YFSRHS, existing national policies on provision of YFSRHS, and youth’s perspectives on these services.

**Findings:**

A total of 23,400 studies were identified through database search and additional 5 studies from other sources. After the full-text screening, 20 studies from 7 countries met the inclusion criteria and were included in the final review. Structural barriers were the negative attitude of health workers and their being unskilled and individual barriers included lack of knowledge among youth regarding YFSRHS. Facilitators of utilisation of the services were mostly structural in nature which included community outreaches, health education, and policy recommendations to improve implementation of the quality of health services and clinics for adolescents/youth to fit their needs and preferences.

**Conclusion:**

Stakeholder interventions focusing on implementing YFSRHS should aim at intensive training of health workers and put in place quality implementation standard guidelines in clinics to offer services according to youth’s needs and preferences. In addition, educating the youth through community outreaches and health education programs for those in schools can facilitate utilisation and scale up of the service.

**Supplementary Information:**

The online version contains supplementary material available at 10.1186/s12978-021-01183-y.

## Plain language summary

Access and utilisation of Youth-friendly sexual and reproductive health is still a big challenge for the youth especially in sub-Saharan Africa. In this study, we explored the underlying reasons for the low access and utilisation of youth-friendly sexual and reproductive health services and potential solutions to the problem.

Articles used in this study were retrieved from different data sources and those that contained barriers and facilitators of access and utilisation of youth-friendly sexual and reproductive health services implementation were summarised.

The key barriers were negative attitude of health workers and their being unskilled emanating from the administrative section theme. The individual factor was the lack of knowledge among youth. The promoters of utilisation were community outreaches, health education and improvement of the quality of services in the clinics for adolescents/ young people’s needs.

Moving forward, stakeholders should aim at increasing the training of health workers and improving the quality of services being offered to the youth. To address the individual barriers, youth should be reached with information through community outreaches and education in schools.

**Supplementary Information:**

The online version contains supplementary material available at 10.1186/s12978-021-01183-y.

## Background

In many African countries, sexual and reproductive health (SRH) needs of young people / youth are often underserved and underestimated despite their demonstrated need and the urgency of these services [1]. Continental population remain high at approximately 1.2 billion with the highest number being youth aged 15–24 years, 226 million—19% of the global youth population—of whom live in sub-Saharan Africa [2]. The term young people which according to the World Health Organisation (WHO) are persons aged between 10 and 24 years and youth (15–24 years) are interchangeably used but often meaning the youth, adolescents, and young people [3]. Youth is characterized as a period of optimum health with a series of physiological, psychological, and social changes that may expose them to unhealthy explorative sexual behaviour such as early sex engagement, unsafe sex and numerous sexual partners and represent 25% of the world population [4, 5].

SRH comprises a major component of the global burden of sexual ill-health. Nearly a quarter of girls aged 15–19 years are married with an estimated 16 million adolescents giving birth each year globally, 95% of whom are from low- and middle-income countries (LMICs) [6]. Trends in delayed marriages do not indicate a decrease in the age of onset of sexual activity among the young people but rather highlights the need to improve access to SRH information, skills and improve services to learn more about sexuality and prevent unwanted pregnancies and sexually transmitted infections [7]. Several factors are contributing to high adolescent/youth fertility rates in sub Saharan Africa, including lack of SRH knowledge, limited access to/use of contraceptives, condoms, and SRHS, gender inequality and cultural practices such as child marriage and initiation ceremonies [8].

In sub-Saharan Africa, adolescents face many significant SRH challenges such as limited access to youth-friendly services (YFS) including information on growth, unsafe abortion, gender-based violence, sexuality, and family planning (FP). This has led youth into risky sexual behaviour resulting in high STI and HIV prevalence among young people, early pregnancy, and vulnerability to delivery complications resulting in high rates of death and disability [6]. Numerous surveys in LMICs indicated that only 33% of young men and 20% of young women have comprehensive knowledge of HIV but still less than half of young men and women surveyed reported using condoms at their last time of sexual activity [8]. According to the 2016 gaps report by UNAIDS, only 10% of young men and 15% of young women were aware of their HIV status which leaves a big challenge to achieving good reproductive health and wellbeing for all [2]. Young girls less than 19 years who get pregnant have a 50% increased risk of stillbirths and neonatal deaths, as well as an increased risk for preterm birth, low birth weight, and asphyxia which in turn affect the health of the unborn child and perpetuate the cycle of poverty [5].

Youth-friendly services are an amalgamation of health facility characteristics, health service provision techniques, and health services offered which are key strategies for improving the health of adolescents in Africa. According to the WHO guidelines, in order to be considered Youth Friendly Health Services (YFHS), the services are required to be accessible, acceptable, equitable, appropriate and effective, gender-equitable and serve as a channel for access to FP and SRH [9]. In 2015, WHO/UNAIDS published the Global standards to improve quality of health-care services for adolescents and ever since then, many countries have adopted and adapted the Global Standards. Although there has been the momentum of implementing SRH services, there are major gaps among the youth in receiving information, the effectiveness of the YFS and skills that are affected by culture, and governmental and financial policies [10, 11]. Youth Friendly Services are a key strategy for improving young people’s health, however, there is an increasing need to break down the barriers to implementation of Youth Friendly Sexual and Reproductive Health Services (YFSRHS) that prevent the young people from accessing quality SRH services in sub Saharan Africa [12]. This study thus aimed at reviewing articles on factors influencing access to and utilisation of YFSRHS in sub-Saharan Africa.

## Methods

### Protocol

The protocol for this systematic review was developed following the Preferred Reporting Items for Systematic Reviews and Meta-Analysis Protocols (PRISMA-P) guidelines for reporting systematic reviews (Additional file 1) [13]. The protocol of this review was registered on PROSPERO (CRD42020173073).

### Data search

Studies were screened to identify those that examined the availability of YFSRHS and youth perspectives on these services used to document the barriers to access and facilitators of utilisation of YFRHS. The electronic journals and reports were searched comprehensively by using PubMed, Web of Science, EMBASE, Medline, Cochrane Library, and Google Scholar databases. Other sources were identified through scanning of references of selected sources. All databases were well-established, multi-disciplinary research platforms, holding a wide variety of peer-reviewed journals, and those that will be kept up to date (Additional file 2).

### Inclusion criteria

The researchers only included studies that were published containing articles from sub-Saharan Africa published from January 2009 to April 2019 and had qualitative and/or quantitative methods and mixed methods. Qualitative research studies included those that employed focus group discussions, in-depth interviews, and structured observations. Quantitative research studies of designs were randomized control trials, cross sectional and case–control. Youth (aged 15–24 years) along with adolescents (10–19) years, were included in this review. The review included studies on youth-friendly service scale-up, utilisation, and access to YFSRHS and were published in English.

### Exclusion criteria

Studies or evaluations carried outside sub-Saharan Africa, multiple publications, systematic reviews or narrative reviews, letters to the editor, case reports were excluded from the review. Articles written in other languages than English were also excluded. Studies with participants predominately greater than 24 or less than 10 years of age or with unclear ages were excluded. Some studies used non-youth key informants and hence excluded.

### Screening

Title and abstract screening of all papers identified by the search strategy were independently performed by two researchers with reference to the published inclusion/exclusion criteria. Key themes were compiled for each article and these themes were grouped based on common traits for thematic synthesis, the result section of each article was analysed using line by line coding. Each category was designated a colour code blue for included and red for excluded. Initial screening of abstracts and titles was done using a process of semi automation while Rayyan QCRI software [14] allowed incorporating a high level of usability. Reference management software Mendeley was used to organise articles retrieved from the comprehensive literature review and then analysed.

## Quality assessment and appraisal of retrieved articles

Quality assessment is crucial to ensure that the findings of the papers are correct and accurate. All studies that meet the eligibility criteria were assessed for quality independently and in duplicate. The included studies were appraised critically for methodological quality and rigour using the Critical Appraisal Skills Programme checklist (Additional file 2) [15]. We used a modified appraisal tool to critically assess the trustworthiness and relevance of the published papers with a keen focus on the study design, sampling methods, participant recruitment strategy, ethical consideration, data analysis, and findings.

### Data extraction

A common data extraction tool was used for all studies, with variation depending on the research design. The extraction included: what information is to be collected on each study (e.g. author, publication source, year), participants and demographics, study design, outcomes, analyses used, and key findings, how the databases or forms was used, how information was recorded and the number of reviewers. Two data extractors (NLR and NR) resolved the discrepancies and any remaining differences were resolved by the other team member (IKC). As part of the extraction process, each qualitative and quantitative study was assessed for methodological rigour. The retrieved data was analysed to answer the main research and specific objectives.

### Synthesis

Finally, the findings were summarized in a narrative synthesis. The synthesis is presented in the results and discussion chapter.

## Results

**Fig. 1:**
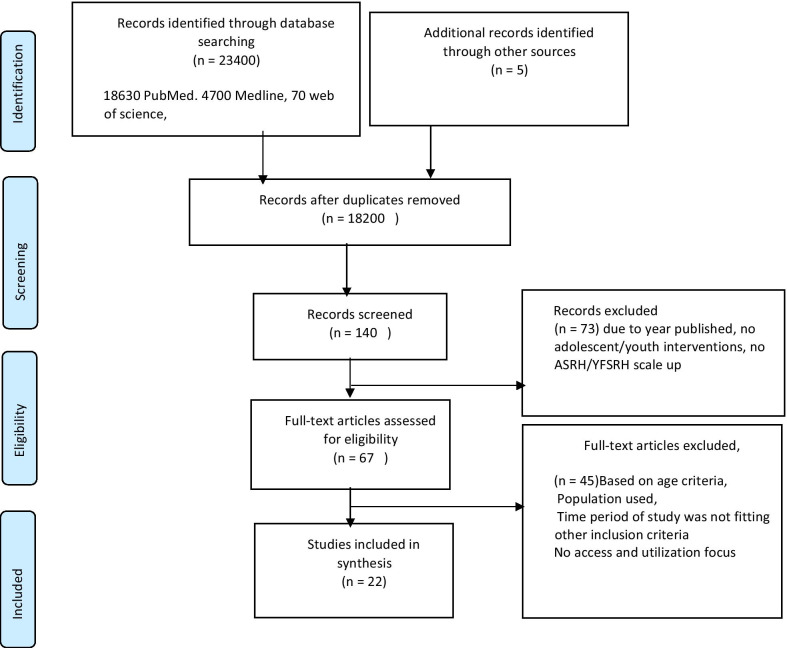
(PRISMA) flow chart: selection process for a systematic review on the access and utilisation of youth friendly sexual and reproductive health in Sub Saharan Africa

A total of 23,400 studies were identified through a database search and an additional five studies from other sources. After the full-text screening, 20 studies met our inclusion criteria (Fig. 1) and were selected for final review*.* We identified studies focusing on access, utilisation and scale-up of A/YFSRHS conducted in sub-Saharan Africa and found articles from 7 countries (Tanzania, Nigeria, Ghana, Kenya, Ethiopia, Uganda, and South Africa) which were included. Nineteen studies used cross-sectional study design, nine selected studies from (South Africa, Kenya, Uganda and Ethiopia) used qualitative, six studies from Nigeria and Ethiopia used quantitative methods and the remaining six studies from Ethiopia, Nigeria, Tanzania and Kenya combined both methods in their studies. Eleven studies had their participants from the community; four studies were done among both rural and urban communities, one study among urban and peri-urban communities and one study in urban communities. In addition, seven studies used participants from health facilities and two recruited participants from schools.

Nineteen articles focused on both males and females and one focused on only females (Table 1).

### Study quality

The studies presented in (Table 1) had varied methodological quality. All the studies had clear aims, objectives, and well-justified rationale. The Critical Appraisal Skills Programme checklist was used to assess for quality of the 20 studies. Of these, 14 studies were of high quality, 4 of medium quality, and 2 of low quality. All studies defined their research design [12, 16, 17]. All studies described their sample size and participants ‘recruitment strategy, though one study adopted a sampling strategy that was deemed inappropriate in relation to the study aims and objectives [18]. The method used for both quantitative and qualitative studies aimed at purposively recruiting participants with rich information on the topic of interest. It was also not clear whether biases were considered during the design of the study and analysis of the data. The following section synthesizes findings on access and utilization of YFSRH interventions in sub-Saharan Africa settings by main YFSRH outcome.

### Barriers to effective access of implementation of youth-friendly sexual and reproductive health services

The barriers to access to YFSRHS were categorized as structural, individual, socio-economic, and socio-cultural. Individual barriers refer to a people having incomplete or incorrect knowledge of SRH, including myths and misconceptions around contraception; limited self-efficacy and individual agency; constrained ability to navigate internalized social and gender norms; and lack of access to information about what SRH services are available and where to seek services [1] structural barriers refer to laws and policies requiring parental or partner consent, distance from facilities, costs of services and/or transportation, long wait times for services, inconvenient hours, lack of necessary commodities at health facilities, and lack of privacy and confidentiality [1]. Cultural barriers which refer to as restrictive norms and stigma around adolescent and youth sexuality; inequitable or harmful gender norms; and discrimination and judgment by communities, families, partners, and providers [1]. Social economic barriers is general term for pressure that prevents people born into lower class from moving over the course to receive better SRH like those from affluent class [1].

#### Individual barriers

The study identified fourteen studies whose primary aim was to evaluate Individual barriers such as knowledge, individual perception, shame and stigma affecting YFSRHS. Studies evaluating the utilisation level of adolescents/ YFRHS found that only (38.5%) adolescents in South Africa and (21.5%) in Ethiopia were knowledgeable about the type of YFSRH services offered [1, 17]. Youths who lacked knowledge of the type of adolescents and YFRHS were not likely to utilize the service than their counterparts [5, 19, 20]. High-quality studies assessing knowledge as a barrier in Nigeria and Ethiopia found that more than two thirds (79.5%) in Lagos, (98.1%) in Port Harcourt, both in Nigeria and (67.3%) in primary health care facilities (Ethiopia) of youths did not know of a specific A/YFRHS provided in their health care facilities [17, 20–24].

Although there YFRHS existed, most adolescents/youths were not aware of these services. According to a medium quality health facility, a cross-sectional study done in Kenya on young people’s perception, knowledge of younger girls (12–14 years) was limited with a majority reporting that they did not know much about condoms, however, boys the same age were more knowledgeable and reported that young people used condoms for prevention of HIV, pregnancy and other STI [25]. According to the multivariable analysis on utilisation factors limiting the youths from accessing YFSRHS, in Ethiopia, those with good knowledge of the type of A/YFSRHS were 1.68 times more likely to utilize A/YFRH service [AOR = 1.68 (95% C.I.: 1.06–2.65)] [19].

Individual perception, fear, shame and stigma affected the utilisation of YFRHS among youth which had a negative impact among those who believed that YFS can improve their health. Youth with stigma and fear about YFSRHS were less likely to utilize the service than their counterparts in a study carried out in Kenya [12]. However, in a study from Tanzania, the youth reported that adolescents do not seek formal treatment for reproductive health problems as a result of shame and fear of disclosure because of the way they will be looked at by the community [19].

A study done in Ethiopia found that participants had the fewest misconceptions about SRH and the most outstanding being misconceptions about oral contraceptive pills causing illness and sterility compared to Rwanda [26]. A study in Malawi also revealed young people’s misconceptions about contraceptive methods. One study participant said “For us youth, there are [contraceptives] which we can take, and there are others which we cannot take as they can bring problems on our lives. The youth mainly use condoms, that one cannot bring problems unlike methods like IUD. People even fall sick because of such methods.” (Female, in-school, 15–17 years, Machinga) [27].

### Structural barriers

Eighteen studies in the review indicated structural barriers affecting the delivery of YFSRHS. High-quality studies from South Africa and Ethiopia addressed primarily provider attitudes and the clinical environment as barriers to adolescents’ access to healthcare during a focus group discussion, however, perceptions of provider attitudes towards adolescents appeared to be inconsistent [22, 28]. During a KI a nurse stated, ‘There are mean nurses but there are good nurses [too]… It’s unfortunate that the South African public, it’s like every time when they go to the clinic they meet the mean nurses only. They never get to meet the good nurses.’ (Female clinical nurse, SSI 4) [28]. Negative attitude of health workers as per the case in one of the studies indicated that 30% had negative attitudes towards the youth in Ethiopia [15]. From focus group discussions (FGDs) in a study done in Uganda, (18/20) participants indicated that experiencing health care provider’s negative attitudes towards providing SRH services affects the utilisation aspects among adolescents [29]. Health worker attitudes can also significantly hinder adolescents’ utilisation of Reproductive Health Service (RHS). Services need to be provided in a youth-friendly environment with health workers that are welcoming and supportive towards adolescents seeking care [30].

At the same time, the number of skilled health workers to offer these services is limited which was identified in a study carried out in South Africa, Ethiopia, and Uganda [16, 31, 32]. The studies indicated the most common barriers to providing health services to young people, and YFS specifically was related to shortages of staff with training on the provision of YFRHS and the lack of a dedicated space for young people at the facilities [20, 22, 33]. Data collected in Tanzania indicated that 37.2% of the service providers who were interviewed reported that they had received training in adolescent sexual and reproductive health (ASRH) information and counselling which is significantly very low and had disparities [12]. Counsellors in a study done in South Africa stated that they had received limited or no training in counselling adolescents. While all counsellors had general HIV/AIDS counselling skills, only a few had received formal training in adolescent development [28].

Many operational barriers in health facilities also impact access and utilisation of these services, such as inconvenient operating times, lack of transportation, and high cost of services [5, 21, 26]. A study in Uganda indicated that the overall quality of SRH services at the facilities was of poor quality to most of them as reported in fifteen of twenty FGDs [29]. In a study from Ethiopia, one of the participants indicated the lack of separate youth clinics saying, designated space for provision of YFSRHS has been mentioned numerous times as a barrier. Even where youth clinics exist, participants report a lack of privacy for SRH services and/or sense of belonging. “When you go to hospitals for services, you may meet your parents there. I remember my friend who met her mother in a clinic” [34].

### Cultural barriers

Four studies were identified exploring the impact of religious and traditional beliefs on access to YFSRHS [21, 23, 26, 34]. Social-cultural factors were greatly associated with some services mainly FP, voluntary counselling and testing, and counselling services. It was established that some cultures and parents in a community cross sectional study done in Kenya and Ethiopia prohibited the youth from utilising YFRHS as this was brought out when a descriptive, chi-square and odds statistics all showed significant relationships [21, 23]. Some participants in a study done in Malawi indicated that parents expressed negative opinions of youth using FP and parents could prevent youth from accessing FP services and also said youth below age 18 are not old enough to be sexually active. Therefore, the youth did not need FP and should focus on completing their education and not engage in sexual activities [26].

### Socio-economic barriers

Three studies reported that adolescents and young people mostly preferred low cost or no charges at all when seeking SRH services from youth centers. A high-quality study exploring barriers and perspectives of youth seeking FP services found that in one district participants some government providers charged fees for FP for both male and female youth. The other mentioned barriers were transport costs and long distances [26]. Similarly, another high-quality study in Uganda [29] and medium quality studies in Kenya [33] and Nigeria [20] also showed similar results as in nineteen of the twenty FGDs, adolescents noted that where the services were not free, the cost was not affordable to them. Two studies in different states of Ethiopia, most respondents mentioned the challenge of cost of services (21%) and (41.2%) respectively, lacked money as its needed to travel to health facilities as the distance/time taken is costly [23, 24].

## Facilitators to the effective utilisation in the implementation of youth-friendly sexual and reproductive health services

The studies included in this review only reported structural facilitators which are described below.

### Community outreach and involvement

Five studies reported on community outreach and involvement in terms of outreach activities in the community, schools and churches among the youth/adolescents. However, some indicated lack of information regarding different areas of YFSRH which was documented in the above studies. A medium quality study done in Ethiopia indicated that (45.9%) had information about the availability of services in the nearby facility and the most important sources of information were peers (54.6%), parents (27.1%), and mass media (7.6%) [19]. The use of local radio stations, posters, magazines, sporting activities and entertainment were mentioned by majority of the respondents in the study as a great way to promote YFSRH [35]. In studies done in Uganda, participants in the outof-the school male adolescent FGDs preferred services such as outreaches in the communities at no cost and preferably with health workers not from the same area [34]. In Malawi a study on youth perspective on how to increase awareness noted that: “outreaches is what will help them [young people] because most of them do not know about what [service] is at the youth centre the youth do not know what kind of youth-friendly [services] are available” (FGD Boys, Meru) [29].

In a study done in Ethiopia, mass media messages (70.9%), advice from others (31.1%), illness of close relative (8.6%) and death of close relative 23(9.4%) were the most important factors that influenced the study participants to utilize the services [19]. Similarly, results from a study in Nigeria indicated that community mobilization for awareness creation and support on SRH issues (59.3%), supported youth to better access SRH services in Primary Health Care Facilities [17].

### School health education

Four studies reported adolescents and young people mostly preferred in-school health education [5, 16, 32, 36] however, some preferred out-school health education as sources of seeking YFSRH services [32]. School health education promoted youth awareness and involvement in access and utilisation of YFRHS as it was indicated in a high-quality study [36]. Participants described health education and specific space for the teenagers as key components of a teenage friendly service with a significant number from a study done in (81.7%) Nigeria said that in-school clubs can create demand for SRH services and 64.7% of them also agreed that out-of-school clubs are important for SRH services [16, 32]. In a low-quality study in Ethiopia, the majority of the respondents (72.7%) who were involved in the available school clubs and (54.3%) had discussed on YFSRH issues with friends put them at high levels of utilisation [5].

Youths who participated in peer to peer discussions were more likely to know about and utilize sexual and reproductive health services than those who did not participate. Peer influence remains a strong factor as shown in this study where peers or friends were found to be the major source of information. Peers were mentioned as resources to support other youth if they shared news and information about FP, but they were also reported to sometimes mock and tease others who they knew wanted to use FP [26]. Friends/peers (45.7%) were the best sources of information on A/YFRHS, however, the most popular services known were FP (81.6%), voluntary counselling and testing (73.8%), and sexually transmitted diseases (67.3%) [21]. The consensus opinion was that young people who came to the Youth Centre to play games or be involved in other activities eventually would end up using the centre’s SRH services if needed [25]. Both girls and boys noted that games such as the pool only attracted boys and made girls shy away from coming to a youth centre. Also, youth playing games at the same place where health services are provided can be a promoting factor as it brings people together to discuss the problems they face and improve them [22, 34].

## Recommendations/options for improving YFSRHS implementation

### Improving the characteristics of YFSRHS to favor youth’s needs and preferences

Two studies indicated how youth’s needs and preferences are to be considered in order to improve YFSRH services. In a high-quality study [28], participants expressed the need for improvement in A/YFSRHS.

Recommendations on the implementation of healthcare service provision should be characterized by a prompt, entertaining and welcoming environment that would encourage adolescents to interact freely. In high-quality study [32], health workers viewed a teenager-friendly service as one that could provide privacy and sufficient time and patience when dealing with teenagers. They also described that a friendly service would be offered by health workers with specific training in teenage pregnancy and with knowledge of how to allocate specific time to teenagers [22]. A study in Nigeria [28] indicated that a large percentage (80.0%) of the respondents believed youth counsellors were best at serving other youth in the community because they are able to relate to their health needs better. In a hospital-based cross-sectional study done in South Africa, one of the respondents in an FGD said; ‘Include teenagers in the programmes. I think that would make a major, major difference.’ (P5 female counsellor) during the design and implementation of the programmes being delivered [17].

In two high-quality studies done both in Uganda [26] and Malawi [29], the most common suggestion among youth participants and parents was the need for more information on FP through counselling which would ensure youth understand the importance on FP and how methods work. A medium quality study in South Africa encouraged training and on-going support to be provided to facilitate this; the importance of such training was to encourage more than one member of staff per facility to be equipped to allow for staff turnover [1]. In Kenya, majority of the respondents wished to see an increase in SRH services especially in rural areas including the use of mobile clinics.

The consensus was that providing a wide range of SRH services in either integrated health facilities or youth centres was more likely to ensure anonymity and that privacy could be maintained [25]. Meeting these standards could make a major contribution to securing adolescents' health, especially in preventing unintended pregnancies and HIV [18].

### Implementing quality standards for YFSRHS

Two high-quality studies assessed another key factor in development and implementation of quality standards found in Tanzania [16] during the scale-up of YFSRHS, and utilisation of YFRHS in Nigeria [24] and recommend that a useful means of ensuring that efforts to make health services adolescent friendly are grounded in wider public health initiatives at the national, regional and council levels.

## Discussion

This systematic review aimed at synthesizing evidence on barriers and facilitators affecting access and utilisation of YFSRHS together with recommendations to improve and scale-up these services for youth/adolescents in sub Saharan Africa. The most common barriers in the review were structural which included the negative attitude of health workers, inconvenient hours, quality of services and unskilled health workers. The health workers attending to the youth were reported to use abusive languages while others were not sympathetic enough to provide services like FP and contraceptives. Moreover, some were not trained adequately/not at all on how to deliver the services to the youth posing a great challenge. A similar observation was found in a context analysis assessing young people’s experience of SRH in sub-Saharan Africa [37].

The review showed the second prominent barrier were at the individual level emanating from limited access to YFSRHS including limited knowledge and awareness among adolescent/youth about the services which is a key hindrance. Adolescents have limited and, in some cases, no access to SRH education and contraception, making adolescent girls more prone to early and unintended pregnancies [38]. To summarize, the youth’s lack of knowledge on YFSRH issues; access to reproductive health information is often hindered because of many different factors including stigma related to young age, parental consent, access to YFSRH services and commodities is challenging because of distance, costs, and quality of services. The studies in this review show similar findings with a systematic review done on SRH knowledge, experiences and access to services among refugee, migrant and displaced girls and young women in Africa which indicated the limited SRH knowledge and awareness among adolescent girls which cause the adolescents to refrain from using them [39].

Few studies reported on socio-economic and cultural barriers due to the fact that some services were not free and the youth lacked money. Others findings from this study indicate that health workers or fellow peers and parental consent on FP services is not given even when these services are offered free. Some services are not free of charge such as FP and the cost of receiving them due to distance is costly, so the youth opted-out from using them. These barriers are due to the context and structure of the environment in which the youth live in.

Only two studies were identified focusing on scale-up of YFS which were from one country (Tanzania) and still had scale-up challenges in the selection and retention of trained health workers and was limited by various contextual factors and structural constraints which still pose a barrier to utilisation of YFSRH [16]. In addition to research on delivering and scaling up YFSRHS to different youths, we should also consider implementation research in different sub-Saharan countries like YFSRHS being grounded in wider public/global health initiatives at the national and regional levels in order to play a larger role in implementation and delivery than in static settings where nongovernmental organizations deliver most of the services.

The review indicated that facilitators to the utilisation of YFSRHS included community outreaches and involvement, school health education, peer-led education and mass media campaigns, and sporting activities and entertainment activities at youth centres which were sources of information preferred by the youth and improved YFHRS access and all were structural in nature. The World Health Organization (WHO) review on universal access showed that actions to make SRHS user friendly and welcoming had led to an increase in the use of services by adolescents [21]. The review suggests that youth are more likely to seek sexual health information from community outreaches and health education in schools and among peers. The health workers' attitude and limited skills should be assessed critically and prioritized as adolescents/youth are willing to access these services through them.

YFSRHS whether offered in dedicated youth centers or public health facilities attract both male and female clients around the world. Similar findings to a study done in Sweden, which has youth centers throughout the country, liberal attitudes and few legal barriers to service provision, however, the majority of patient visits to youth centers were made by females [40].

This review identified the need to improve access to and standardise the quality of health services for adolescents/youth needs along with integrating efforts such as educate, empower and support adolescents. A user-friendly SRHS does not necessarily ensure service utilization by adolescents/youth. Similarly, a review done on assessing YFSRHS indicated the need for standardisation and prioritisation of indicators for the evaluation of YFSRHS which include accessibility, staff characteristics and competency, and confidentiality and privacy favoring youth’s needs [2]. During the scale-up of YFSRHS in Tanzania, there were gaps in the standardisation of services according to Global standards for quality of health-care services for adolescents which is still a major challenge. Standardized systems within a country on the use of data recorded at the health facility level and combined supportive supervision with regular self-assessments to improve the quality of services is a facilitator to utilisation of YFSRHS which has not been found in any articles reviewed hence a gap. The Global Accelerated Action for the Health of Adolescents (AA-HA!): guidance to support country implementation recommends that standards-driven quality improvement should be positioned within national adolescent health programmes within a specific country [2]. Despite the existence of laws and policies, effective implementation can only be managed through political commitment, adequate resource allocation, capacity building and the creation of systems of accountability to cater for effective access and utilisation of YFSRHS [3]. Evidence shows that focusing on strengthening health systems to meet the adolescents’ needs has a positive effect on access and uptake of some YFSRHS [41]. Further, evidence shows that many health system interventions and reforms have led to an increase in coverage of several health services [11]. These gaps point to the need for robust and timely research on the mechanisms through which YFSRH facilitators can increase utilisation and access across a variety of sub-Saharan Africa. Further studies should be done on how cultural factors such as religion and beliefs affect access and utilisation of YFSRH services.

Evidence on attribution is particularly weak, with majority of studies using a cross-sectional design, with no control group. Qualitative studies have the potential to contribute rich perspectives from study populations on YFSRH service utilisation and barriers to access, but we found only three studies using this design, and six studies using mixed methods to assess YFSRH. Overall, only 65% of the studies (n=13) selected were graded as high quality, 30% as medium quality (n=6), and 5% as low quality (n=1). There was limited number of use of stratification, by gender and age as some studies indicated the differences, and so we were not able to capture potentially differing health access and utilisation outcomes among adolescents/youth.

In terms of limitations, the narrow inclusion criteria may have led to the exclusion of some peer-reviewed literature and conference articles. Additionally, our language inclusion criteria, i.e. only studies published in English, imposed by the capacity of the research team may have limited the numbers of hits returned by our search and led to publication bias. Nevertheless, this review provides important information on barriers and facilitators of access and utilisation of YFSRHS implementation and proposes key recommendations which should inform design and implementation of effective YFSRHS programmes.

## Conclusion

The review has shown that most common barriers impeding YFSRH services were due to structural barriers such as the negative attitude of health workers and unskilled health workers, and individual barriers emanating from low levels of knowledge among the youth/adolescents. Regarding facilitators of utilisation, results showed that with sustained community involvement and outreach, school health education, recreational activities, and the provision of free or reduced-cost YFSRH to those with a financial constraint, there will be an increase in utilisation together giving the youth access to the health services hence promoting sustainability. The Global guidelines on standardisation of health services encourage that adolescent service providers prioritise quality however, YFSRHS are highly fragmented, poorly coordinated and uneven in terms of quality. Pockets of excellent practice exist, but, overall, services need significant improvement and should be brought into conformity with existing guidelines. The review emphasizes the need to educate and health train the youth/adolescent to know more about the reproductive health services being provided at youth-friendly centers and their involvement in the design and implementation of interventions targeting them. Stakeholder interventions focusing on implementing YFSRHS should aim at intensive training of health workers and put in place quality implementation standard guidelines in clinics to offer services according to youth’s needs and preferences.

**Table 1:** characteristics of included studies exploring barriers to access and facilitators of the utilisation of youth-friendly sexual and reproductive health services among the youth

Authors name and year	Country	Study settings	Study design	Aim and objective	Approach	Age and sex	Findings	CASP quality assessment
Mulaudzi et al. 2018 (50)	South Africa	Hospital	Cross sectional	To explore barriers to providing adolescent friendly sexual and reproductive health services	Focus group discussion and semi structured interviews	Both female and male	*Barriers*; health care providers attitude, Counsellors reported inadequate training to address adolescent psychosocial issues, including adolescents-specific ages as counsellors	High quality
Godia et al. 2014 (47)	Kenya	Health care facilities and youth centers	Cross sectional	Understanding of the SRH problems young people face and document perceptions of available SRH services as reported by young people themselves. explored experiences and perceptions of young people	Focus group discussion and indepth interviews	15–24 boys and girls	*Barriers;* in their responses were broad and reflect the cultural, social and economic environment in which they live *Facilitators;* Recreational activities attract the boys. Increasing awareness through outreaches	Medium
Helamo et al. 2017 (42)	Ethiopia	Institutions	Cross sectional	Assesses factors affecting adolescents and youths friendly reproductive health service utilisation among high school students in Hadiya zone, Ethiopia	Quantitative	15–24 years female and male	*Barriers;* Youths with a good knowledge of the type of A/YFSRHS were more likely to utilize the service than their counterparts, utilisation levels were low and youth were unaware of the services being provided	Medium
Ajike et al. 2016 (44)	Nigeria	Rural and urban	Cross sectional	The knowledge of youths on available adolescent/youth friendly services (A/YFRHS) in Ikeja, Lagos State, Nigeria	Quantitative	15–24 years boys and girls	*Barriers;* The participants knew what adolescent/youth friendly services were but did not know where to get these services from because they were not aware of the available A/YFRHS facilities	High quality
Self et al. 2018 (48)	Malawi	Community	Qualitative	To explore the perspectives of youth and adults about the drivers and barriers to youth accessing family planning and their ideas to improve services	Focus group discussion	15–24 years female and male	*Barriers;* to youth accessing family planning included contraception misconceptions, the costs of family planning services, and negative attitudes. Parents had mixed views on FP,	High quality
Atuyambe et al. 2015 (51)	Uganda	Urban and peri urban	Qualitative	To assess the sexual reproductive health needs of the adolescents and explored their attitudes towards current services available	Focus group discussions	10–24 years male and female	*Recommendations*; establishing adolescent-friendly clinics with standard recommended characteristics (sexuality information, friendly health providers, a range of good clinical services such as post abortion care	High quality
Chandra-Mouli et al. 2013 (39)	Tanzania	Urban and rural	Survey	To extend the reach of Adolescent Friendly Health Services (AFHS) in the country	Qualitative	15–24 years female and male	*Barriers;* poor knowledge, it had received reports that the quality of the AFHS being provided by some organizations was poor	High quality
							*Recommendations/policy;* standardized definition of AFS	
Zewdie et al. 2018 (49)	Ethiopia	In schools	Cross sectional	Young people’s perceptions and barriers towards the use of sexual and reproductive health services in Southwest Ethiopia	Focus group discussion	15–24 years female and male	*Barriers*; poor perceptions about SRH, feeling of shame, fear of being seen by others, restrictive cultural norms, lack of privacy, in available services	High quality
Rukundo et al. 2015 (52)	Uganda	Community	Cross sectional	Views concerning factors affecting availability, accessibility and utilization of teenager friendly antenatal services in Mbarara Municipality, southwestern Uganda	Key informant interviews	15–19 years female and male	*Barriers*; health workers described their experience with teenagers as challenging due to their limited skills when it comes to addressing adolescent-specific needs	Medium
Eremutha et al. 2019 (40)	Nigeria	Rural and urban areas	Stratified and purposive	To generate increased understanding of the barriers that limit youth access to sexual and reproductive health services(SRH) offered by Primary Health Care (PHC) facilities in Nigeria	Mixed method	10–24 female and male	*Facilitators*; community mobilization for awareness creation and support on SRH issues will support youth to better access *Barriers*; lack of awareness, negative attitude of health workers, cost of service and parents perception or fear	High quality
Betebebu Mulugeta et al. 2019 (53)	Ethiopia	Facility based	Cross sectional	To assess youth-friendly service quality and associated factors at public health facilities in Arba Minch town, Southern Ethiopia	Quantitative	15–19 female and male	*Facilitators;* comfort and providers sex, waiting time, place of YFS, are factors which are significantly associated with client satisfaction in a health facility	High quality
Ayehu et al. 2016 (43)	Ethiopia	Community	Cross sectional	To assess young people’s sexual and reproductive health service utilization and its associated factors in Awabel district, Northwest Ethiopia	Quantitative	15–24 years male and females	*Facilitators;* Young people from families of higher family expenditure, lived with mothers, participated in peer education and lived near to a Health Center were more likely to utilize SRHS at youth centers	High quality
Binu et al. 2018 (6)	Ethiopia	School based	Cross sectional	To assess utilisation of Sexual and Reproductive Health (SRH) services and its associated factors among secondary school students in Nekemte town, Ethiopia	Quantitative	10–24 years female and male	*Barriers;* Inconvenient times, lack of privacy, religion, culture, and parent prohibition were barriers to SRH service uptake cited by the school youths	Low
James et al. 2018 (35)	South Africa	Health facilities	Cross sectional	To detail the evaluation of AYFS against defined standards to inform initiatives for strengthening these services	Qualitative	15–24 years male and female	*Barriers;* Facilities had the essential components for general service delivery in place, but adolescent specific service provision was lacking especially the sexual and reproductive health services	Medium
Geary et al. 2014 (41)	South Africa	Rural health facilities	Survey	Investigate the proportion of facilities that provided the Youth Friendly Services programme and examine healthcare workers’ perceived barriers to and facilitators of the provision of youth friendly health services	Qualitative	12–24 years female and male	*Barriers*; lack of youth-friendly training among staff and lack of a dedicated space for young people, health workers attitude, did not appear to uphold the right to access healthcare independently. breaches in young people’s confidentiality	High quality
Motuma et al. 2016 (45)	Ethiopia	Community	Cross sectional	to assess the extent of youth friendly service utilization and the associated factors among the youth	Mixed methods	15–24 years female and male	*Barriers*; source of information and having knowledge about services were associated with utilisation, negative perception about counselling affected the outcomes	High quality
Renju et al. 2010 (13)	Tanzania	Health facilities	Survey	A process evaluation of the tenfold scale up of an evaluated youth friendly services intervention in Mwanza Region, Tanzania, in order to identify key facilitating and inhibitory factors from both user and provider perspectives	Mixed methods	15–24 years female and males	*Barriers*; scale up faced challenges in the selection and retention of trained health workers and was limited by various contextual factors and structural constraints	High quality
Obonyo Perez Akinyi 2009 (24)	Kenya	Community	Cross sectional	Examined how those factors determined or affected the utilization patterns of YFRHS by the youth. mitigating and addressing challenges to scale up	Mixed methods	10–24 years female and male	*Facilitators;* level of education, type of school and youth’s awareness about existence of reproductive health facility and services offered were significantly associated with utilization	Medium
Chimankpam Williams Uzoma 2017 (46)	Nigeria	Health facility	Cross sectional	To assess the utilization of youth friendly health services by young people in Port Harcourt and factors that affect utilisation	Mixed methods	15–24 years female and males	*Barriers*; low knowledge levels *Facilitators*; Friends/family/contemporary and notice board were major sources of information	High quality
Berhe et al. 2016 (54)	Ethiopia	Community	Cross sectional	Assess utilization of youth-friendly services and associated factors in Mekelle city	Mixed methods	15–29 years females and males	*Barriers*; negative attitude towards youth friendly service utilization *Facilitator*; awareness and prior knowledge were predictors of utilisation	Medium

Numbers in brackets in this table are corresponding number of articles retrieved from the inclusion criteria

## Supplementary Information

**Additional file 1.** PRISMA 2009 Checklist.

**Additional file 2.** CASP checklist for Quality assessment tool.

## Data Availability

The data that support the findings of this study are from different datasets (e.g. PubMed, Google, Google Scholar, and Medline) and from Malawi Institution’s Library (Journal Section) and are included in the list of references.

## Abbreviations

A/YFSRHS: Adolescent/youth friendly sexual and reproductive health services
SP: Service providers
