# Supplementary material for: Factors influencing access to and utilisation of youth-friendly sexual and reproductive health services in sub-Saharan Africa: a systematic review
Source: Reprod Health. 2021 Jun 27;18:135. doi: 10.1186/s12978-021-01183-y (PMC8237506; doi:10.1186/s12978-021-01183-y)
Supplement: Supplementary file 2 — Additional file 2. CASP checklist for Quality assessment tool. [file 12978_2021_1183_MOESM2_ESM.doc]

**CASP checklist for Quality assessment tool**

| **Questions** | **(50)** | **(47)** | **(42)** | **(44)** | **(48)** | **(51)** | **(39)** | **(49)** | **(52)** | **(40)** | **(53)** | **(43)** | **(6)** | **(35)** | **(41)** | **(45)** | **(13)** | **(24)** | **(46)** | **(54)** |
| --- | --- | --- | --- | --- | --- | --- | --- | --- | --- | --- | --- | --- | --- | --- | --- | --- | --- | --- | --- | --- |
| Is there a clear statement of the aims and research question? | yes | yes | yes | yes | yes | yes | yes | yes | yes | Yes | yes | Yes | yes | yes | Yes | Yes | Yes | yes | yes | yes |
| Is the methodology appropriate for the study? | yes | yes | partly | yes | yes | yes | yes | yes | yes | Yes | yes | Yes | partly | yes | Yes | Yes | Yes | yes | Yes | partly |
| Is the research design appropriate to address the aims of the research? | yes | yes | yes | yes | yes | yes | yes | yes | yes | Yes | yes | Yes | partly | yes | Yes | Yes | Yes | yes | Yes | yes |
| Have ethical issues been taken into consideration? | yes | yes | Yes | yes | yes | yes | yes | yes | yes | Yes | yes | Yes | yes | yes | Yes | Yes | Yes | yes | yes | yes |
| Is the sampling strategy appropriate to address the Authors name, country? | yes | yes | Not clear | yes | yes | yes | yes | yes | partly | yes | yes | Yes | no | yes | Yes | Yes | Yes | yes | Yes | yes |
| Are the method of data collection appropriate and clearly explained? | yes | yes | yes | yes | yes | yes | yes | yes | yes | yes | Yes | Yes | yes | yes | Yes | yes | yes | yes | Yes | yes |
| Is the description of the data analysis sufficiently rigorous and comprehensively described? | yes | yes | yes | yes | yes | yes | yes | yes | partly | yes | Yes | Yes | yes | partly | Yes | yes | yes | partly | Yes | partly |
| Is there a clear description of the findings and results? | yes | partly | yes | yes | yes | yes | yes | yes | yes | yes | Yes | Yes | yes | yes | Yes | yes | yes | partly | Yes | yes |
| Are the findings of the study generalizable or transferable to a wider population? | yes | partly | yes | yes | yes | yes | yes | yes | no | yes | Yes | Yes | no | partly | Yes | yes | Yes | yes | Yes | yes |
| How important are these findings to policy and practice? | yes | yes | yes | yes | yes | yes | yes | yes | yes | yes | Yes | yes | Partly | Yes | Yes | yes | yes | yes | Yes | yes |
| Total | High | medium | Medium | high | high | high | high | high | medium | high | high | high | low | medium | high | high | high | Medium | high | medium |

**All criteria fulfilled = High quality=1*

*Six and above criteria fulfilled =medium=2*

*Less than six criteria fulfilled = Low=3*

SEARCH STRATEGY

| Search | Add to builder | Query | Items found |
| --- | --- | --- | --- |
| #7 | Add | Search **((adolescent) AND (adolescent friendly sexual and reproductive health)) AND sub Saharan Africa** | 64 |
| #6 | Add | Search **((youth) AND (youth friendly sexual and reproductive health)) AND sub Saharan Africa** | 67 |
| #5 | Add | Search **((sub Saharan Africa) OR south part of Africa) OR (low and middle income countries)** | 224286 |
| #4 | Add | Search **(((sexual and reproductive health)) AND youth sexual reproductive health) AND adolescent sexual reproductive health** | 5566 |
| #3 | Add | Search **(((youth friendly sexual) AND reproductive health services) AND adolescent friendly sexual) AND reproductive health services** | 152 |
| #2 | Add | Search **((youth friendly services) OR youth centers) OR adolescent friendly services** | 48649 |
| #1 | Add | Search **((youth) OR adolescents) OR young people** | 2592709 |
